# Peer Interventions for Hepatitis C Testing and Treatment in OECD Countries: A Systematic Scoping Review

**DOI:** 10.1111/jvh.70130

**Published:** 2026-01-23

**Authors:** Sorcha Daly, Leila Reid, Ryan Buchanan, Peter McCulloch, Paul Flowers, Jamie Frankis, Gabriele Vojt

**Affiliations:** ^1^ Hepatitis C Trust London UK; ^2^ Faculty of Medicine University of Southampton Southampton UK; ^3^ National Institute for Health and Care Research (NIHR) Health Protection Research Unit in Evaluation and Behavioural Science University of Bristol Bristol UK; ^4^ Bristol Medical School University of Bristol Bristol UK; ^5^ Department of Psychological Sciences and Health University of Strathclyde Glasgow UK; ^6^ School of Health and Life Sciences Glasgow Caledonian University Glasgow UK

**Keywords:** blood‐borne infections, community health workers, health services, hepatitis C, peer influence, people who inject drugs, scoping review

## Abstract

Services delivered by peer workers (people with lived/living experience) are recommended to find, test, and treat those at risk of hepatitis C (HCV). However, there is a lack of knowledge around the characteristics and underlying mechanisms of existing HCV peer interventions and how these drive effectiveness and impact. This systematic scoping review aimed to identify the activities of peer interventions, their reported outcomes, and mechanisms of change. We systematically searched five databases (Scopus, PubMed, Embase, PsycINFO and Web of Science) for peer‐reviewed papers which described HCV peer interventions in OECD countries published between 2012 and 2022, informed and structured by the PRISMA extension for scoping reviews and scoping review reporting guidance. All identified studies were double screened at title and abstract and full text stage. Twenty‐nine studies met our inclusion criteria. In 23 studies, peer workers delivered interventions, mostly focused on outcomes for intervention recipients. Peer workers improved HCV care linkage, testing, treatment and SVR12 rates. Peer workers themselves reported increased confidence, job satisfaction, improved mental wellbeing, employability and social integration into communities. Key activities and peer intervention elements were occasionally documented, but more often omitted. None of the included studies explicitly documented or theorised underlying mechanisms, that is, how or why peer interventions work. The lack of details and mechanistic descriptions of peer interventions negatively impact the ability to optimise and enhance peer‐led HCV care. This potentially undermines the elimination of HCV at population level.

## 1 Introduction

Hepatitis C virus (HCV) is a blood‐borne virus that can lead to chronic disease, cirrhosis and hepatocellular carcinoma [1]. The advent of direct‐acting antivirals (DAAs) has made HCV elimination feasible, prompting the World Health Organisation (WHO) to set targets for HCV elimination by 2030 [1, 2]. Despite advances in HCV service delivery such as rapid HCV testing and treatment scale‐ups [3, 4], people who inject drugs (PWID) and people with experience of incarceration world‐wide remain disproportionately affected. Marginalised groups (e.g., PWID) face multiple barriers to accessing HCV care resulting in persistent gaps across the HCV care cascade which consists of screening, diagnosis, linkage to care, treatment initiation, adherence and cure [5, 6, 7]. These barriers include low health and HCV literacy which hinder perceived candidacy for HCV care, fear of positive HCV test results, long waiting times between referrals, distrust of healthcare professionals, competing life priorities affecting people's ability to attend set appointments, perceived stigmatisation from healthcare providers and a highly mobile lifestyle due to factors such as unstable housing (see Appendix 1 for a tabularised literature summary of facilitators and barriers across the HCV care cascade steps). Accordingly, addressing inequalities in access and engagement is key to achieving elimination goals. International evidence suggests that community‐based and decentralised HCV service models improve engagement and treatment uptake among marginalised populations [3, 8]. Integration with community, pharmacy, and harm reduction services, co‐location with other healthcare services, and concurrent opioid agonist treatment enhance retention across the care cascade [5, 9, 10]. Alongside these decentralised models, awareness campaigns have shown value in strengthening the HCV care cascade. For example, in the UK, the HepCATT trial [11] demonstrated that raising awareness and facilitating access in drug treatment clinics significantly improved testing, referrals and treatment initiation. Other effective interventions include incentives to improve linkage and treatment initiation, nurse‐ and pharmacist‐led prescribing, and peer‐assisted telemedicine to support adherence and treatment completion, particularly in underserved or remote populations [12, 13, 14]. In addition, coordinated service delivery and data‐driven monitoring further bolster progress towards elimination [10, 15]. Over and above these initiatives, the WHO [16] recommends peer involvement as a key strategy towards elimination. Peer workers with lived/living experience of HCV, substance use and/or marginalisation enhance HCV prevention, testing and treatment uptake and completion [12]. Importantly, the roles and opportunities for peer‐led interventions alongside DAAs have increased (when compared to interferon‐based HCV treatments), especially since integrating assertive outreach approaches into HCV care delivery. Yet, our knowledge of the specific components of peer interventions, what they improve and how they work remains limited. Understanding mechanisms of action is essential to optimise, evaluate and translate HCV peer interventions across settings.

To this end, we conducted a systematic scoping review of the peer‐reviewed literature on HCV peer interventions, identifying intervention activities, outcomes and underlying mechanisms. Our review questions are (1) what do peer workers do?, (2) what do peer workers achieve? and (3) how do peer interventions work?

## 2 Methods

### 2.1 Research Design

We conducted a systematic scoping review following Arksey and O'Malley's guidance [17]. This approach is well suited to collating and mapping the characteristics and outputs from diverse intervention studies. Unlike systematic reviews, scoping reviews do not evaluate the methodological quality of studies, which allows greater flexibility in synthesis across different study types [17]. We report findings according to the PRISMA‐ScR reporting guidelines [18].

### 2.2 Search Strategy

We systematically searched five databases: Scopus, PubMed, Embase, PsycINFO and Web of Science, using free text and controlled vocabulary terms (conducted on August 3, 2022). Herein, we considered peer interventions as a whole, including all aspects of peer involvement (e.g., case management, patient‐centred care), while acknowledging the diversity in peer approaches [19]. Search terms were co‐produced via patient and public involvement and engagement by consulting peer workers delivering HCV peer interventions and cross‐referencing search terms in systematic reviews on HCV peer interventions. We grouped search terms into two conceptual categories referring to: (1) peer interventions (e.g., ‘peer worker’, ‘peer model’) and (2) hepatitis C virus (e.g., ‘HCV’, ‘hep C’). We defined peer workers as people with ‘direct, personal experience of the challenges faced by the target population’ [20]. We extended our definition for this review to include peer workers with lived or living experience, in employed or voluntary roles, who specifically (co‐)deliver an intervention (rather than a research study) to find, test and treat people at risk of HCV. Appendix 2 details our search terms and records returned across each database. We restricted searches to peer‐reviewed publications to enhance study quality, English language and publication date from 2012 (when DAA drug trials began [21]) to 2022. We excluded papers outside OECD countries to focus on interventions within similar socioeconomic environments.

### 2.3 Study Selection

All studies were independently screened by two reviewers (SD, GV) using Covidence software at title and abstract, and full text screening stage. We included studies at abstract and title stage if they described a peer intervention and HCV or blood‐borne viruses (BBV). Any disagreements were resolved through discussion and, where necessary, by a third reviewer (LR) until consensus was reached. Next, we used the inclusion and exclusion criteria (Table 1) to screen the resulting studies at full text. Finally, we expanded our search by manually searching the reference lists of any reviews identified during screening.

**TABLE 1:** Inclusion and exclusion criteria at full text screening.

Inclusion	Exclusion
Peer intervention relating to HCV. Interventions delivered by peer workers, i.e., people with lived or living experience of HCV or other relevant factors (e.g., injecting drug use) to find, test and treat those at risk of HCV. Primary focus and description of peer support for HCV. At least one outcome related to peer interventions. Peer intervention aimed at adults (aged > 18 years). Published, peer‐reviewed journal articles. Primary research studies only. OECD country. Published 2012 onwards. English language. Any study design.	Peer intervention unrelated to HCV. Intervention not described or not delivered by peer workers. Non‐formalised peer network. HCV‐related support or outcomes not separated from otherBBV outcomes. Peer intervention aimed at young people (aged < 18 years). Grey literature. Reviews or other non‐empirical work. Non‐OECD countries. Papers published prior to 2012. Non‐English language.

### 2.4 Data Extraction

We extracted data on study country, setting, design, research question(s), aims and objectives, methods, analysis, outcomes and demographics including sample size, gender, average age and age range of peer workers and intervention recipients (Appendix 3). We also documented descriptions of peer workers (Who are peer workers?), peer interventions (What do peer workers do?), their reported outcomes (What do peer workers achieve?) and underlying mechanisms (How do peer interventions work?).

We defined underlying mechanisms as the reported processes, structures and influences that operate in particular contexts to generate peer intervention outcomes of interest (e.g., peer workers effectively testing and treating people for HCV) [22]. Regardless of whether the intervention was deemed successful (e.g., an increase in HCV testing rates) or not, we sought authors' explanations of how the peer intervention worked/did not work within each article. We focused on information explicitly stated. For example, we looked for theorisation or clear labelling of mechanisms explaining reported outcomes or the described peer intervention. If information was not explicitly stated, then we extracted implicit information where possible (e.g., we considered explanations and descriptions in relation to individual mechanisms and processes underpinning the intervention outcomes from anywhere in the paper).

### 2.5 Data Synthesis

**FIGURE 1:**
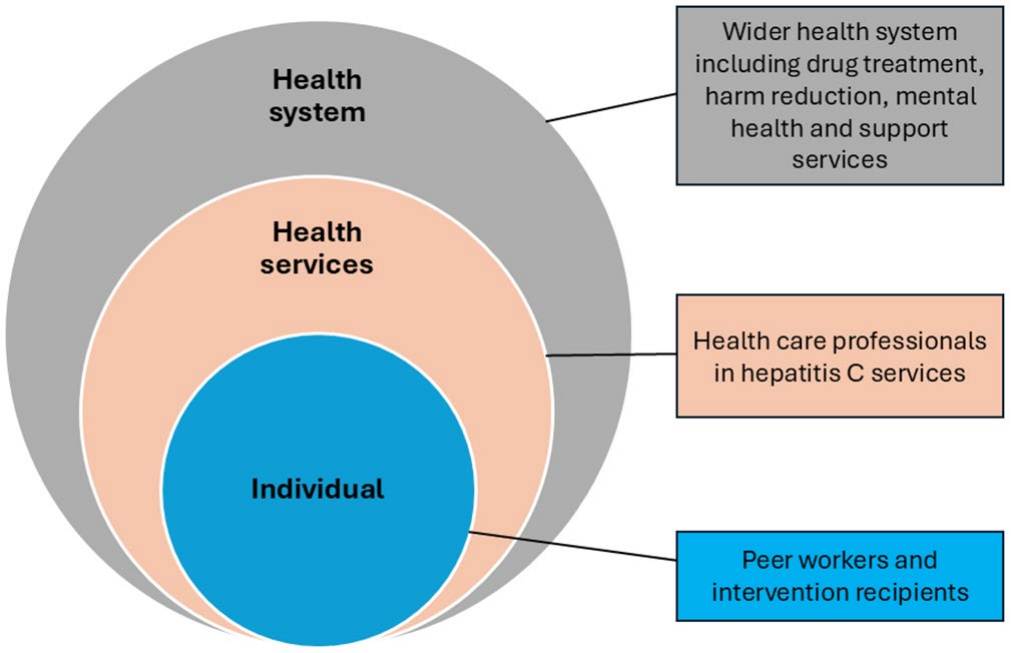
Adapted socio‐ecological framework.

We synthesised findings narratively. Study outcomes were summarised in terms of intervention impacts (or lack thereof). We organised underlying mechanisms through an adapted socioecological lens [23] (Figure 1).

This allowed us to contextualise our mechanistic findings within a multi‐levelled range of socio‐ecological structures. In this way, we considered the implications of our findings on mechanisms at the individual (e.g., peer workers and intervention recipients), service (e.g., local healthcare organisation such as a hospital or drug treatment clinic) and system (e.g., national health system) level. Understanding causal mechanisms across these diverse yet interconnected levels, i.e., the entire system, is associated with effective health improvements and patient outcomes [24].

## 3 Results

**FIGURE 2:**
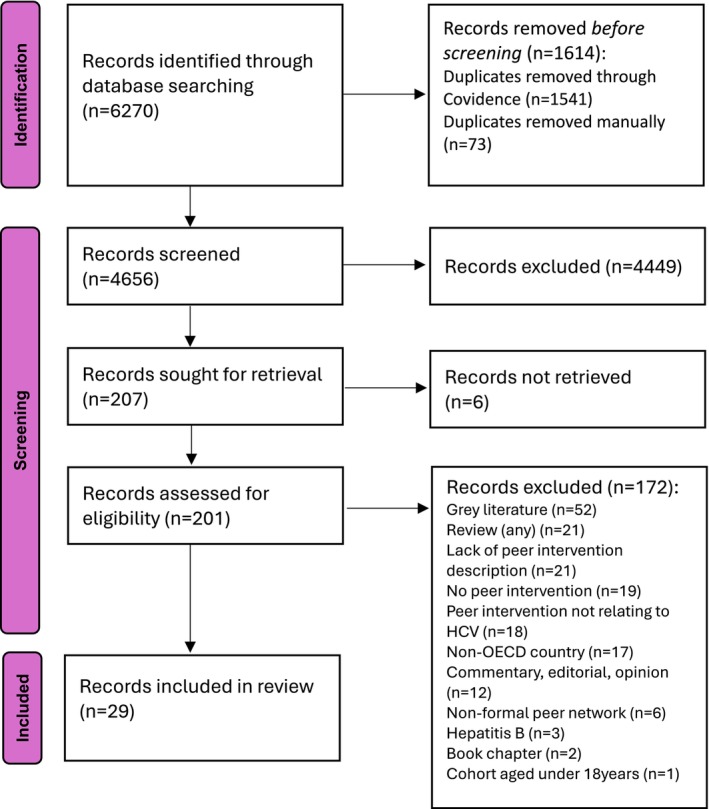
PRISMA diagram.

We identified 6270 records through database searching. After removing duplicates and screening at title and abstract stage, we sought the full text for *n* = 207 records. We excluded *n* = 178 records; our final sample included 29 records. We found no new studies in existing reviews. The full search and exclusion results are reported in the PRISMA diagram (Figure 2).

### 3.1 Characteristics of Included Studies

Twenty‐nine studies met the inclusion criteria for this scoping review [25, 26, 27, 28, 29, 30, 31, 32, 33, 34, 35, 36, 37, 38, 39, 40, 41, 42, 43, 44, 45, 46, 47, 48, 49, 50, 51, 52, 53]. Appendix 3 outlines each included study with detailed information on the data extracted. Here, we summarise study characteristics in terms of year published, study country and setting, design and reported target population in Table 2.

**TABLE 2:** Summary characteristics of included studies.

Study characteristics (*n* = 29)		Number (%)
Country	UK	7 (24)
	US	7 (24)
	Australia	6 (21)
	Canada	3 (12)
	Ireland	2 (7)
	New Zealand	1 (3)
	France	1 (3)
	Norway	1 (3)
	Belgium	1 (3)
Setting	Community – urban	22 (76)
	Prison	2 (7)
	Community—rural	2 (7)
	Community and prison	2 (7)
	Community—rural and urban	1 (3)
Study design	Qualitative	8 (28)
	Quantitative	14 (48)
	Mixed methods	7 (24)
Target population	People who use/inject drugs	19 (66)
	Indigenous communities	4 (14)
	People with experience of incarceration	2 (7)
	Not stated/NA	2 (7)
	‘Hard to reach’^a^ groups diagnosed with HCV	1 (3)
	HCV peer educators^a^ within prisons	1 (3)

^a^ Verbatim from authors' description in included studies.

Most (*n* = 23) studies [25, 26, 27, 28, 30, 31, 32, 33, 34, 37, 38, 40, 42, 43, 44, 45, 46, 47, 49, 50, 51, 52, 53] focused on understanding or evaluating a stand‐alone peer intervention. Eight of these were qualitative or mixed methods, with peer workers acting as research participants by describing their peer work and reflecting on their experience of being or transitioning into the peer worker role [25, 28, 37, 44, 45, 46, 49, 53]. Fifteen studies [26, 27, 30, 31, 32, 33, 34, 38, 40, 42, 43, 47, 50, 51, 52] were quantitative or mixed methods, including randomised controlled trials [27, 31, 43, 50, 51] and described peer intervention outcomes for intervention recipients. The remaining six studies [29, 35, 36, 39, 41, 48] described peer interventions as one part of a larger, multi‐component HCV intervention. For example, this included peer‐delivered support alongside tailored health promotion, HCV telemonitoring and usage of HCV surveillance data [29], co‐locating HCV services with a drug treatment clinic [35] as well as mobilising peer workers to co‐design and develop a patient‐centred community HCV service [41].

There is some overlap in the peer interventions identified. Five studies [26, 32, 44, 52, 53] described the HCV intervention ‘HepCare’ in Ireland [32, 33] and England [15, 44, 52] respectively. Two studies [50, 51] reported on ‘CHAMPS’ (Chronic HepAtitis C Management to ImProve OutcomeS) and two others [30, 47] examined the ‘Deadly Liver mob’ intervention.

#### 3.1.1 Characteristics of Peer Workers

Fifteen studies [25, 28, 29, 30, 31, 32, 37, 39, 41, 42, 43, 44, 45, 46, 49] provided information on a total of 588 peer workers. The median peer sample size was *n* = 7 (range *n* = 2–482). Peer workers were mostly male (median sample proportion 71%, range 50%–100%) with a mean age of 47.5 years (range 26–64 years).

#### 3.1.2 Characteristics of Intervention Recipients

Intervention recipients were people diagnosed with or at risk of HCV, and included populations derived from prison and community settings, including hospitals, drug treatment, needle and syringe programmes, homeless hostels and pharmacies. All included studies with intervention recipients (*n* = 24) reported sample sizes [25, 26, 27, 29, 30, 31, 32, 33, 34, 35, 36, 38, 39, 40, 41, 42, 43, 45, 47, 48, 49, 50, 51, 52]. The median sample size was *n* = 305 (ranging from *n* = 17–30,729). Twenty studies [26, 27, 28, 30, 32, 33, 34, 35, 36, 38, 39, 40, 43, 45, 47, 48, 49, 50, 51] provided further demographic information; most intervention recipients were male (median sample proportion 67%, ranging from 47% to 86%), with a mean age of 43.0 years (range 14–91 years).

### 3.2 Reported Features and Activities of Peer Interventions

Included studies reported the features and activities of peer interventions. Intervention features broadly related to (i) training peer workers, (ii) conducting outreach and engagement, (iii) delivering HCV awareness and education (to both intervention recipients and staff) and (iv) providing emotional, psychological and practical support to intervention recipients undergoing testing and treatment. Peer activities supported service provision both across and beyond the HCV care pathway, including outreach and engagement with wider BBV testing (e.g., hepatitis B), harm reduction (e.g., safe injecting) and signposting to other support services (e.g., mental health services, housing).

#### 3.2.1 Training Provided to Peer Workers

Nineteen studies [26, 27, 28, 31, 32, 33, 34, 37, 38, 40, 42, 44, 45, 46, 50, 51, 52, 53] reported training of peer workers. Training content was detailed in five studies [32, 42, 44, 45, 46], while six provided minimal information [26, 31, 37, 38, 40, 53] and seven did not describe training content other than ‘peers were trained’ [27, 28, 33, 34, 50, 51, 52]. The training provided included life skills (e.g., professionalism and job readiness) [32, 44, 45, 46, 53], ‘self‐care’ (including setting boundaries and confidence building) and communication skills (e.g., public speaking) [37, 40, 42, 44, 46]. Eight studies [26, 31, 32, 38, 40, 42, 44, 45] included HCV skills specific training, which involved HCV‐related testing and assessment (e.g., point of care testing, fibroscan), and five studies [31, 32, 40, 42, 44] described HCV knowledge and awareness (e.g., risk factors, diagnosis, disease progression, treatment pathways, pre‐ and post‐HCV test counselling, advocacy and confidentiality practices). No peer‐to‐peer training or peer mentoring activities were reported.

#### 3.2.2 HCV Outreach and Engagement

Outreach and engagement activities to recruit intervention recipients into HCV testing and treatment programmes were reported in 14 studies [26, 30, 31, 33, 34, 35, 38, 40, 41, 42, 43, 45, 51, 52]. This included peer workers delivering mobile outreach service with a van [38] or attending places where people who use drugs reside and/or meet (e.g., homeless shelters, needle and syringe programmes) [40]. To support engagement, peer workers utilised role modelling and group facilitation (e.g., peer workers facilitating group meetings with intervention recipients) [42]. However, descriptions of peer workers' outreach work content were minimal across studies.

#### 3.2.3 HCV Awareness and Education

Seventeen papers [25, 27, 28, 30, 31, 32, 34, 35, 41, 42, 44, 45, 46, 47, 48, 49, 53] reported that peer workers provided HCV awareness raising and education, that is, giving talks and sharing personal experiences. Of these, only five [25, 30, 41, 45, 47] described the key components of this work, specifically: (i) explaining that information was shared verbally and via demonstration [8], (ii) providing culturally appropriate information [47], (iii) accommodating low literacy [30, 41, 47], (iv) utilising games, group discussions and interactive activities to communicate [45] and (v) ensuring accessibility for all ages and delivering clear messaging around HCV as a preventable and curable disease [41]. Peer workers used self‐disclosure of HCV and other relatable factors (e.g., injecting drug use experience) to engage potential intervention recipients [27, 36, 43, 46, 48, 53].

#### 3.2.4 Testing and Treatment Support

Twenty‐seven studies [25, 26, 27, 28, 30, 31, 32, 33, 34, 35, 36, 38, 39, 40, 41, 42, 43, 44, 45, 46, 47, 48, 49, 50, 51, 52, 53] reported that peer workers helped intervention recipients to engage with HCV testing and peer workers undertook the actual testing themselves in four papers [31, 34, 40, 44]. Emotional and psychological support to access and engage with HCV treatment was described in 11 studies [26, 28, 31, 32, 36, 37, 39, 40, 46, 49, 53]. Peer workers supported intervention recipients to emotionally process their diagnoses, provided reassurance on the effectiveness of HCV treatment [26] and motivated intervention recipients to complete treatment [46]. Practical support to engage with HCV treatment was reported in 16 studies [26, 28, 32, 34, 35, 36, 39, 42, 43, 46, 48, 49, 50, 51, 52, 53]. Detailed descriptions of practical treatment support were rare. Rather, authors briefly noted activities such as collecting and accompanying intervention recipients to HCV appointments [26, 52] or facilitating out‐of‐hours checks on their wellbeing [32]. Three studies [26, 34, 35] reported peer workers supporting intervention recipients post HCV diagnosis.

#### 3.2.5 Additional Intervention Activities

Twelve papers [26, 31, 32, 34, 35, 38, 40, 43, 44, 45, 49, 53] reported additional intervention activities outside of training, outreach and support. These were harm reduction (e.g., advice on safer injecting) [45, 53], advocacy (e.g., peer workers representing intervention recipients in health and social settings), and addressing stigma [26, 35, 44, 49], patient assessment (e.g., documentation of drug use history), and/or research data collection [31, 32, 38, 40, 45], other BBV testing (e.g., hepatitis B) [31, 34, 40, 44], sharing of HCV and/or BBV test results [40], providing support for wider health challenges, and signposting to relevant services (e.g., mental health counselling) [43].

### 3.3 Outcomes of Peer Interventions

Intervention outcomes were measured and documented for both intervention recipients and peer workers. However, none of the included studies explicitly assessed the impact of peer interventions on the health system they work within or the service providers they work with.

#### 3.3.1 Peer Worker Outcomes

Ten studies reported outcomes for peer workers. Seven qualitative studies [28, 32, 37, 44, 46, 49, 53] documented positive personal and professional progress, improved relationships, increased confidence, employability, self‐efficacy and a sense of pride for peer workers. Of the quantitative studies (*n* = 3), outcomes for peer workers were not clearly reported. That is, two [31, 34] implied positive outcomes for peer workers since they had increased their HCV skills (HCV testing procedures) and knowledge, while one study [33] noted there were benefits for the peer workers but failed to report what these benefits were.

#### 3.3.2 Intervention Recipient Outcomes

Twenty‐one studies [27, 28, 29, 30, 31, 32, 33, 34, 35, 36, 38, 39, 40, 41, 42, 43, 45, 47, 48, 49, 50, 53] reported outcomes of peer interventions on intervention recipients. Eighteen studies outlined positive impacts [26, 27, 28, 30, 31, 32, 33, 35, 38, 39, 40, 41, 42, 43, 44, 45, 47, 48, 49]. These were explicitly reported in relation to increased HCV case finding, improved patient engagement and willingness to attend screening, improved HCV knowledge, increased HCV treatment uptake and completion, and increased willingness and attendance at follow‐up appointments, for example, SVR12 testing. Other outcomes were implied in the reporting, for example, increased HCV prevention, improved health literacy, increased local community awareness and improved health service attendance.

Mixed outcomes were reported by two studies [34, 36]. In a qualitative study [36], intervention recipients considered peer interventions as both a barrier and facilitator, depending on personal preference and need (e.g., in terms of the intervention recipient's perception of recovery). Further, a UK wide quantitative study [34] noted that although peer workers improved HCV testing uptake when implemented, their positive effect had not increased at 2 months post implementation.

Negative outcomes were reported in only one qualitative study [53]. Intervention recipients stated the peer intervention did not meet their expectations as peer workers had lived rather than living experience of HCV treatment and were not independent but governed and shaped by a healthcare organisation.

#### 3.3.3 Outcomes Reported for Health Services

None of the included studies focused explicitly on outcomes for health services or healthcare professionals. Instead, the conclusions from two studies [35, 49] implied positive impacts. Peer workers were thought to have freed up HCV nurse specialists' time [35] and optimised treatment decision making among healthcare professionals by representing the patient perspective [49]. In one study [47], healthcare professionals expressed concerns over workforce capacities to support peer workers.

#### 3.3.4 Outcomes Reported for the Health System

Two studies [29, 52] suggested that peer interventions were cost‐effective when compared to standard care. One study [39] implied that peer worker involvement in a community‐based ‘test and treat’ setting might have been crucial to the overall HCV intervention success, which in turn was estimated to be cost‐effective.

### 3.4 Underlying Mechanisms

None of the included studies explicitly reported mechanisms. We therefore summarise underlying mechanisms implicitly noted by authors, which attempt to explain how peer interventions work. We structured these at the individual, health service and system level (see Figure 1) [25].

#### 3.4.1 Individual Level Mechanisms

Underlying mechanisms were not reported explicitly. Thirteen studies [26, 28, 30, 35, 36, 37, 42, 44, 45, 46, 47, 48, 49] implied the importance of peer workers' shared lived and/or living experience, not just of HCV but of marginalisation and adversity in general. This facilitated rapport and trust with intervention recipients and underpinned peer workers' commitment and motivation to engage intervention recipients. These studies also reported the importance of individual peer workers' abilities to negotiate the interface between *street* and *institution* effectively, to build connections through explicit confidentiality, to address power imbalances in health and cultural settings, and to normalise the personal experience of HCV and navigating through the health system. Three studies [26, 32, 42] stressed the value of training peer workers to increase confidence and competence, in particular when conducting HCV testing. However, none attempted to theorise, label, or explain these individual mechanisms.

#### 3.4.2 Service Level Mechanisms

None of the included studies explicitly documented service‐level mechanisms. Instead, 16 studies [25, 28, 29, 31, 34, 35, 36, 37, 38, 41, 42, 44, 46, 47, 52, 53] discussed a range of service‐level barriers and/or facilitators supporting the implementation and positive outcomes of peer interventions. In particular, studies implied the importance of an organisational commitment to a harm reduction philosophy [34, 53], an inclusive and non‐stigmatising work ethos [34, 44], and flexible service delivery (e.g., drop‐in, mobile, co‐located and independent service models) [8, 31, 35, 38, 41, 47, 52]. Similarly, resourcing service infrastructures to include peer workers within healthcare services (e.g., as part of multidisciplinary meetings) [28, 29, 36, 42, 44, 46] was reported to support effective interventions. Three studies [37, 46, 53] outlined the importance of implementing professional mentorship programmes, and one study [44] advised training and payment routes for peer workers delivering the intervention.

#### 3.4.3 System Level Mechanisms

System‐level mechanisms were not explicitly reported. However, four studies [38, 41, 46, 47] implied that effective peer interventions required supporting the credibility of peer workers across health care teams [41, 47], establishing patient advisory boards [46], and streamlining care and access to HCV treatment [38].

### 3.5 Integrative Synthesis

**FIGURE 3:**
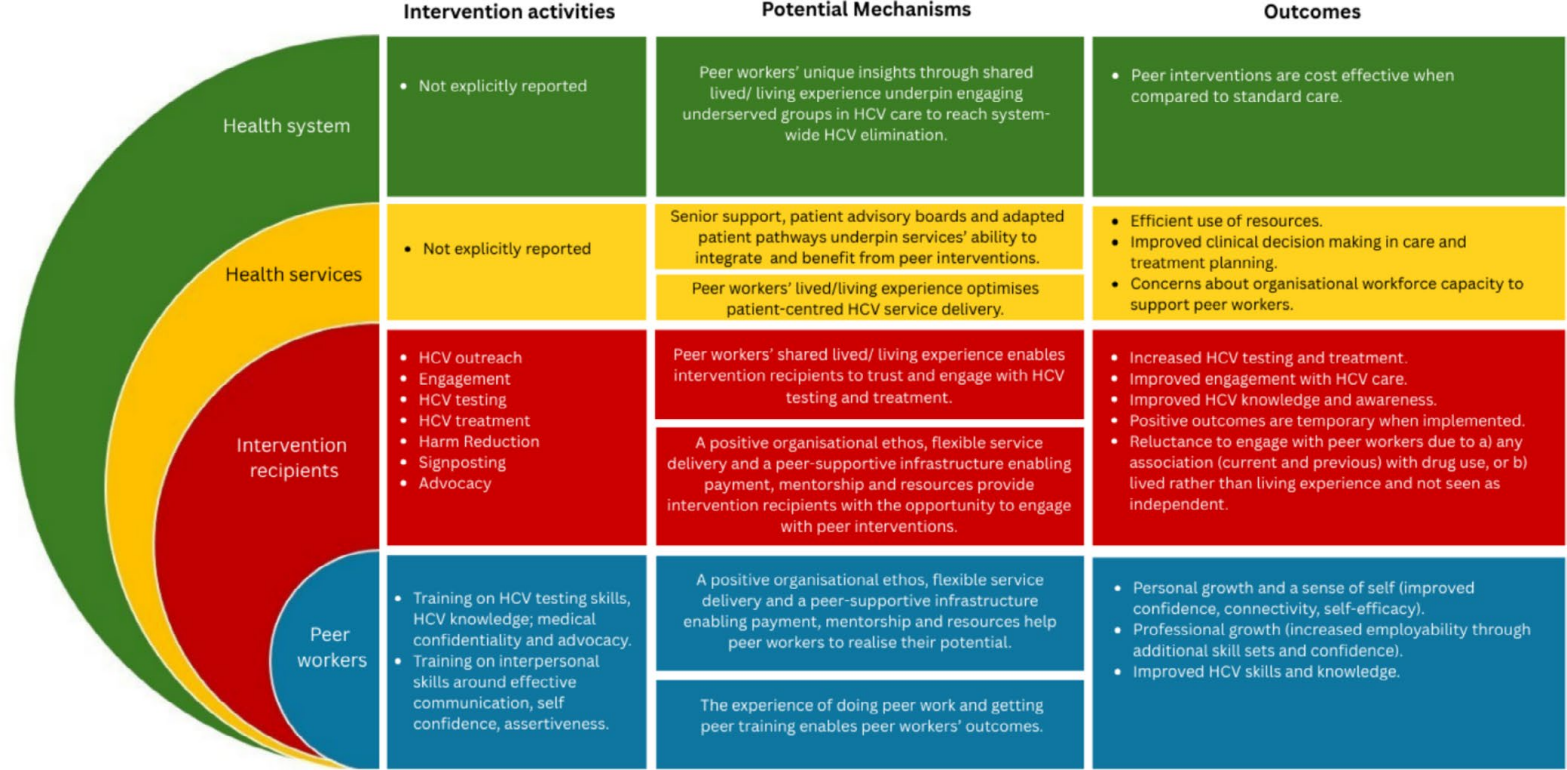
Integrated synthesis map of included literature on HCV peer interventions: Intervention activities, outcomes and mechanisms.

We consolidated our review findings by mapping the intervention activities and outcomes of peer interventions across different socioeconomic levels (Figure 3). We also highlighted the possible (albeit implicit) mechanisms of change underpinning these outcomes, linking these back to the interventions' identified activities. Critically, our findings suggest that peer workers' shared lived or living experience underpinned changes and outcomes at all levels across McLeroy et al.'s [23] socio‐ecological model.

## 4 Discussion

This scoping review examined the activities, outcomes and underlying mechanisms of HCV peer interventions described within the academic peer‐reviewed literature. We identified 29 heterogenous studies reporting multiple peer intervention approaches, which exemplified the diversity of the literature. Most studies highlighted the positive impacts of peer interventions for both intervention recipients and peer workers themselves, for example, upskilling peer workers and directly enhancing HCV case finding and healthcare engagement. In qualitative studies, peer workers were described as being able to build trust, support and respect via their shared lived/living experience of adversity, substance use, HCV and the wider healthcare system. This allowed them to bridge gaps between healthcare service providers and intended intervention recipients. In quantitative studies, peer workers were associated with improved short‐term outcomes such as increased HCV testing rates and treatment uptake. Our findings, especially when visualising intervention activities, outcomes and (implicit) mechanisms to peer interventions, suggest that peer workers operate at all socio‐ecological levels and across the HCV care cascade, thereby changing the environment in relation to HCV awareness and stigma, service‐level design and accessibility, and individual access to care and self‐efficacy. However, peer interventions were inconsistently described within the existing literature, with few details of intervention features and activities provided, with limited descriptions of who peer workers were, how they worked, or what training and skills they had. When peer interventions and activities were described, these tended to cluster around the individual levels (i.e., peer workers and intervention recipients) rather than the service and system levels (see Figure 3). Clear theorisation describing how the peer intervention worked was missing from all studies, yet this is crucial for programme implementation, evaluation and scale‐up. As shown in our findings and visualisations, this lack of clarity around mechanisms of action is interlinked to an absence of system‐ and service‐level guidance detailing how to integrate peer interventions into existing healthcare services.

### 4.1 Comparison to Existing Literature

These findings are in line with existing reviews which have shown positive outcomes of peer interventions for both peer workers and intervention recipients within HCV (12), other chronic conditions, for example, diabetes [54] and asthma [55], and other blood‐borne viruses (e.g., HIV [56, 57]). Included economic studies suggest that despite being expensive, peer interventions seem to be cost‐effective when compared to standard HCV care, however further research is required. This echoes reviews of peer‐led interventions for other health related behaviours [58] and mental health [59]. The lack of clear reporting including explicit labelling and theorisation of underlying mechanisms in peer interventions has also been found in reviews of other health conditions. For example, King and Simmons [19] highlight that peer interventions in mental health tend to focus on intervention recipients, omitting impacts and changes at the social and system level. This suggests that work to address these issues is required not just around HCV care interventions, but in health intervention development and evaluation work more broadly.

### 4.2 Implications

The implications of this review point to a need for more systematic descriptions of peer intervention content, mechanisms and adequate theorisation to enable optimisation and transferability of peer interventions across different contexts. A deeper understanding of how context, content, mechanisms and outcomes interact is needed to understand and subsequently enrich and improve HCV care delivered by peer workers. While some of the included studies implicitly suggest that social influences, achieved via shared lived/living experience of HCV, marginalisation and disadvantage, are an important feature of the peer intervention, this alone is unlikely to be sufficient nor the sole mechanism underpinning the effectiveness of peer interventions. Rather, peer interventions are complex [26, 32] consisting of dynamic and interacting intervention elements, which are further influenced by contexts and changes, for example, within the wider health system and at service levels (see Figure 2). Therefore, research, evaluation and reporting on peer interventions need to accommodate this complexity, identifying how the intervention is, or is not, ‘acceptable, implementable, cost effective, scalable and transferable across contexts’ [60]. Utilising a complex intervention framework enables the remit of peer intervention research and evaluation to incorporate broader definitions of complexity and include the development and identification of interventions as they are situated and contributing to change within whole systems. This facilitates more detailed and relevant reporting and supports the identification of *context‐specific* underlying mechanisms influencing intervention outcomes. This will then enable resources and efforts in public health to focus on effective and efficient interventions and their components in eliminating HCV.

### 4.3 Future Research

Given the range of interventions documented in our review, hybrid‐effectiveness implementation trials (type 2, type 3) or implementation trials with embedded process evaluations of diverse peer interventions and models to identify what works best for whom, under what circumstances and how would be useful. Realist approaches that facilitate deductive identification of outcomes and impacts may be beneficial for future evaluations of peer interventions, enabling the identification of the *context‐specific* underlying mechanisms that influence intervention outcomes. Further research is required to better understand peer workers as a group of professionals and defining ‘peerness’ (i.e., what makes a peer a peer) in practice and social processes. Longitudinal work on the cumulative effects of peer interventions could benefit from exploring (possibly) long‐term behavioural changes, at the intervention recipient, peer worker and health service level. This could aid our overall understanding of the inputs and adaptations of patient pathways required to support peer interventions at the service and health system level.

### 4.4 Strengths and Limitations

Strengths include utilising structured frameworks for scoping reviews in addition to incorporating aspects of the PRISMA scoping review guidance, thus ensuring a rigorous approach. We implemented a systematic search strategy and included the input of peer workers with lived experience in defining search terms and focus. The review also benefitted from a multidisciplinary team approach, that is, third sector working directly with peer workers, academic researchers and input from a UK research advisory committee including HCV epidemiologists, practitioners and researchers. In terms of limitations, we may have missed important publications by excluding non‐English language papers and papers from non‐OECD countries, possibly negatively affecting the diversity and insights of our findings. Due to limited resources, we did not include grey literature; we did not contact any authors for supplementary information on the peer interventions or additional relevant publications, and we did not use a pre‐set framework (e.g., theoretical domains framework) to code implicit descriptions of mechanisms.

## 5 Conclusion

The role of peer workers in supporting and changing individual and social HCV‐related factors strengthens research and practice, emphasising the importance of structural and individual‐level interventions to reduce health inequalities and exclusion. However, the features of peer interventions for HCV care are inadequately reported and do not explicitly document underlying mechanisms. This limits knowledge and understanding for the scalability, credibility and value (including cost‐effectiveness) of peer‐led HCV care and its contribution towards HCV elimination. Complex intervention frameworks should be used to ensure consistent and detailed reporting, which in turn supports changes, implementation and optimisation of healthcare at health service and system level.

## Funding

This work was supported by the Hepatitis C Trust.

## Conflicts of Interest

The authors declare no conflicts of interest.

## Data Availability

The data that support the findings of this study are available in Appendix 2 (search strategy) and Appendix 3 (data extraction table, listing all included studies by author, country, setting, design, research aims, sample, focus, peer intervention features and activities and peer intervention outcomes).

## Appendix 1 Summary of Steps in the HCV Care Cascade and Associated Barriers and Facilitators in Literature

**Table:**

HCV cascade step	Barriers	Facilitators
Screening/Diagnosis	Low HCV awareness Stigma (drug use, incarceration, HCV) Limited access to testing Fear of positive results	Point‐of‐care/rapid testing Integration into routine care, harm reduction, prisons Peer outreach and education Public health awareness campaigns
Linkage to Care	Complex referral pathways Long wait times Distrust of healthcare providers Competing priorities (housing, mental health, substance use)	Patient navigation/case management Peer support and advocacy Decentralized care (community clinics, pharmacies) Simplified referral processes
Treatment initiation and adherence	Multiple appointments Restrictive eligibility criteria Concerns about side effects Financial/insurance barriers Stigma from providers Co‐existing substance use or mental health issues	Simplified single‐visit assessments Task‐shifting to nurses/non‐specialists Non‐invasive liver testing methods Short, well‐tolerated DAA regimens Integrated addiction/mental health services Peer support to encourage adherence Flexible, low‐threshold delivery models
Treatment Completion & Cure (SVR)	Loss to follow‐up (unstable housing, incarceration) Reinfection risk Limited post‐treatment monitoring	Active follow‐up and re‐engagement Peer/community support networks Education on reinfection prevention and liver health

## Appendix 2 Search Terms

The following search terms were entered into 5 databases listed below. The words ‘peer’ and ‘consumer’ were omitted from the agreed search terms as their inclusion resulted in unmanageable numbers of papers being returned.

**Table:**

Identification Date	Database	Search Terms	Results without screening for date	Results with screening for date range, language and peer reviewed.	
03.08.2022	Scopus	hcv OR “HEP C” OR “Hepatitis C” AND “Peer Suppor*” OR “peer led” OR “peer advoca*” OR “peer based” OR “peer intervention*” OR “peer coach*” OR “peer deliver*” OR “peer speciali*” OR “peer mentor*” OR “peer partner*” OR “peer model*” OR “community health worker” OR “CHW” OR “support broker” OR “peer provid*” OR “peer service*” OR “peer counsel*” OR mentor* OR “lived* experience*” OR “living* experience*” OR “social network*” OR “social network support*” OR “recovery all*” OR “lay health work* “ OR “LHW” OR “community health volunteer*” OR chv OR “peer progra*” OR “Peer Educa*” OR “peer mode*” Note: go to advanced search and cut and paste the entire search terms into the query string	5195	4087 (limited to date and language) no option for limiting to peer review. And then broken down by year: 2020–2022 = 1560 2019–2016 = 1520 2015–2112 = 1007	4087 Imported to endnote 5 Aug 2022 in date batches
03.08.2022	PubMed	hcv OR “HEP C” OR “Hepatitis C” AND “Peer Suppor*” OR “peer led” OR “peer advoca*” OR “peer based” OR “peer intervention*” OR “peer coach*” OR “peer deliver*” OR “peer speciali*” OR “peer mentor*” OR “peer partner*” OR “peer model*” OR “community health worker” OR “CHW” OR “support broker” OR “peer provid*” OR “peer service*” OR “peer counsel*” OR mentor* OR “lived* experience*” OR “living* experience*” OR “social network*” OR “social network support*” OR “recovery all*” OR “lay health work* “ OR “LHW” OR “community health volunteer*” OR chv OR “peer progra*” OR “Peer Educa*” OR “peer mode*” Note: Immediately go to advanced search, enter Peer search terms in Query box, go to Actions, click on ‘add query’, then enter HCV search terms and hit search	320	243 (no option to select for peer review on Pubmed?)	243 exported to endnote on 5 August 2022
3/08/2022	Embase	(used the multi field search option) hcv OR “HEP C” OR “Hepatitis C” AND “Peer Suppor*” OR “peer led” OR “peer advoca*” OR “peer based” OR “peer intervention*” OR “peer coach*” OR “peer deliver*” OR “peer speciali*” OR “peer mentor*” OR “peer partner*” OR “peer model*” OR “community health worker” OR “CHW” OR “support broker” OR “peer provid*” OR “peer service*” OR “peer counsel*” OR mentor* OR “lived* experience*” OR “living* experience*” OR “social network*” OR “social network support*” OR “recovery all*” OR “lay health work* “ OR “LHW” OR “community health volunteer*” OR chv OR “peer progra*” OR “Peer Educa*” OR “peer mode*”	619	498 (limited for date and language but no option to limit for peer reviewed article only)	498 exported to endnote on 5 August 2022
03/08/2022	PsycINFO	(used the multi field search option) hcv OR “HEP C” OR “Hepatitis C” AND “Peer Suppor*” OR “peer led” OR “peer advoca*” OR “peer based” OR “peer intervention*” OR “peer coach*” OR “peer deliver*” OR “peer speciali*” OR “peer mentor*” OR “peer partner*” OR “peer model*” OR “community health worker” OR “CHW” OR “support broker” OR “peer provid*” OR “peer service*” OR “peer counsel*” OR mentor* OR “lived* experience*” OR “living* experience*” OR “social network*” OR “social network support*” OR “recovery all*” OR “lay health work* “ OR “LHW” OR “community health volunteer*” OR chv OR “peer progra*” OR “Peer Educa*” OR “peer mode*”	1747	1142 (limited to date and language ) 1049 (limited for date, language and peer reviewed only)	1049 exported to endnote on 5 August 2022
03.08.2022	Web of Science	hcv OR “HEP C” OR “Hepatitis C” AND “Peer Suppor*” OR “peer led” OR “peer advoca*” OR “peer based” OR “peer intervention*” OR “peer coach*” OR “peer deliver*” OR “peer speciali*” OR “peer mentor*” OR “peer partner*” OR “peer model*” OR “community health worker” OR “CHW” OR “support broker” OR “peer provid*” OR “peer service*” OR “peer counsel*” OR mentor* OR “lived* experience*” OR “living* experience*” OR “social network*” OR “social network support*” OR “recovery all*” OR “lay health work* “ OR “LHW” OR “community health volunteer*” OR chv OR “peer progra*” OR “Peer Educa*” OR “peer mode*”	526	393 (limited to date and language) no option to limit to peer review only	393 exported to endnote on 5 August 2022

## Appendix 3 Characteristics of Included Studies

**Table:**

References	Author (year)	Country	Setting	Design	Research aims relevant to peer intervention	Sample	Focus	Peer intervention features/activities	Outcomes
[25]	Newland & Treloar (2013)	Australia	Community	Mixed methods	To examine a self‐determined peer education programme conducted by a drug user organisation.	**Peer workers:** *n* = 17, male *n* = 9 (53%), mean age 42 years, age range: 27‐48 years. **Intervention providers:** *n* = 26, no demographics reported.	Peer workers as main intervention Peer workers as research participants	HCV Education (patients), harm reduction.	Peer workers reported to engage with clients about safer injecting techniques (39%, *n* = 1234/3373 logged peer activities), HCV discussions (37%, *n* = 1154/3373 logged peer activities) and other life issues, e.g., overdoses, new drugs and risks (24%, *n* = 760/3373 logged peer activities). Qualitatively, peer workers reported effective peer education via verbal information and demonstration. Being linked to a drug user organisation sometimes enhanced credibility. Peer workers reported to act as advocates for clients (e.g., to address stigma).
[26]	Surey et al. (2019)	UK	Community	Quantitative	To describe the role of peer support in linking marginalised individuals to specialist treatment services.	**Peer workers:** not reported. **Intervention recipients:** *n* = 197, male 79%, mean age 46 years, age range not reported.	Peer workers as main intervention	Treatment support, incentives, emotional support, maintaining engagement, advocacy.	Peer workers facilitated HCV care linkage and HCV treatment uptake among marginalised groups of people. Of people referred to secondary care and peer worker support, 52.8% (*n* = 104/176) engaged with HCV treatment centres. Of these, 85.6% (*n* = 89/104) started HCV treatment and of these, 85.4% (*n* = 76/89) were cured. [No comparison group]
[27]	Arain et al. (2016)	Belgium	Community	Quantitative (RCT)	To assess the influence of combining formal education, peer education and FibroScan assessment on knowledge and willingness for HCV screening and treatment among PWUD attending opioid substitution treatment programme.	**Peer workers:** not reported. **Intervention recipients:** *n* = 52; Male *n* = 40 (77%), mean age 39 years, age range not documented.	Peer workers as main intervention	HCV education and experience sharing through self‐disclosure (patients).	The peer intervention statistically increased HCV knowledge in patients (*p* < 0.0001). Willingness to HCV testing and treatment uptake were descriptively higher in the intervention group at 1 month (60% vs. 86%) and 3 month (67% vs. 77%) follow‐up. [no statistical *p* value reported]
[28]	Batchelder et al. (2017)	USA	Community	Qualitative	To analyse patients' perceptions, both peer educators and non‐peer educators, of an HCV peer educator programme.	**Peer workers:** *n* = 13 **Intervention recipients:** *N* = 17 Combined demographics reported. Male *n* = 20 (66%), mean age 51 years. Age range not documented.	Peer workers as main intervention Peer workers as research participants	HCV education (patients), treatment support, advocacy, emotional support.	Peer workers facilitate patient engagement and HCV treatment completion by providing [themes] ‘emotional support’, ‘myths and fears dispelled’, ‘experiences are normalised’, ‘living examples of treatment success’. Peer workers reported benefits to be [themes] ‘perceived as experts’, and engaging in ‘pro‐social behaviour’.
[29]	Behrends et al. (2019)	USA	Community	Quantitative	To present cost estimates for Project INSPIRE from a provider perspective. INSPIRE [Innovate and Network to Stop HCV and Prevent complications via Integrating care, Responding to needs and Engaging patients and providers] combines HCV surveillance data, case identification with individual‐level care coordination, peer support, health promotion, adherence support, HCV tele‐mentoring.	**Peer workers:** *n* = 7, no demographics reported. **Intervention recipients:** *n* = 987, no demographics reported.	Peer workers as minor part of larger HCV intervention	Training, conducting case conferences.	The HCV care coordination intervention [INSPIRE] including peer workers was cheaper and more beneficial when compared to free federal health provision (Medicare) in the US per patient per care episode ($66–93 vs. $164). [no statistical *p* value reported]
[30]	Biggs et al. (2016)	Australia	Community	Quantitative	To assess whether the peer‐based, incentive‐driven Aboriginal ‘deadly liver mob’ intervention increased attendance and STI/BBV screening.	**Peer workers:** *n* = 2, no demographics reported. **Intervention recipients:** *n* = 306, male *n* = 144 (47%), mean age 32 years, age range 14–91 years.	Peer workers as main intervention	HCV education, Indigenous community, outreach and recruitment.	The peer intervention statistically increased the number of Aboriginal people attending HCV testing (as part of STI/BBV testing) (*p* < 0.01) and HCV testing uptake when compared to previous testing without peer workers (70 vs. 23%); (*p* < 0.001).
[31]	Broad et al. (2020)	Canada	Community	Quantitative (RCT)	To evaluate the feasibility of using peer outreach workers to deliver POC HCV antibody testing.	**Peer workers:** *n* = 11, no demographics reported. **Intervention recipients:** *n* = 380, male *n* = 260 (68%), mean age 43 years, age range not reported.	Peer workers as main intervention	HCV education, outreach, testing, antibody diagnosis, emotional support, POC test administration, data collection.	Peer‐led outreach POC testing improved HCV care engagement when compared to other outreach models. There was no significant difference in the number of people who presented for HCV care follow up between the two intervention groups (POC with peer workers vs. TAU) (*n* = 6, 3% vs. *n* = 5, 3%) (*p* > 0.05).
[32]	Connolly et al. (2021)	Ireland	Community and prison	Mixed methods	To provide an overview of the methods of each component of HepCare Ireland. [HepCheck, HepLink, HepFriend, HepEd, HepCost]	**Peer workers:** *n* = 12, no demographics reported. **Intervention recipients:** *n* = 812, male *n* = 700 (86%), mean age 43 years, age range not reported.	Peer workers as main intervention	HCV education (patient), emotional support, data collection, treatment support.	As part of a multi‐level HCV intervention, HepFriend facilitated HCV testing (98.3%, *n* = 798/812), HCV RNA identification (35.1%, *n* = 280/798), HCV care linkage (58.6%, *n* = 164/280), HCV treatment completion (56.7%, *n* = 93/164) and achieved SVR12 (55.9%, *n* = 52/93). [No comparison group]
[33]	Crowley (2019)	Ireland	Prison	Mixed methods	To report the feasibility and impact of a peer‐supported HCV screening and linkage‐to‐care intervention to increase the numbers of HCV infections detected.	**Peer workers:** not reported. **Intervention recipients:** *n* = 425, male *n* = 425 (100%), mean age 33 years, age range not reported.	Peer workers as main intervention	Promotion, testing support, recruitment, awareness raising.	The peer intervention facilitated HCV case finding and screening (12% in the study population, *n* = 50/419) including 5% (*n* = 19) untreated HCV infections. [No comparison group]
[34]	Jugnarain et al. (2022)	UK	Community and prison	Quantitative	To review the impact of peer support implemented alongside HCV healthcare service delivery in England.	**Peer workers:** not reported. **Intervention recipients:** *n* = 30,729, male *n* = 16,641 (71%), mean age 49 years, age range not reported.	Peer workers as main intervention	HCV education (patient and staff), testing, treatment support, incentives (food, drink, travel), engagement and recruitment.	The inclusion of peer workers in HCV service delivery increased patient referrals and HCV treatment uptake in drug services (OR = 2.45; 95% CI, 1.49–3.84) (*p* < 0.01). There was no statistical increase in patient numbers testing and completing HCV treatment at 2 months follow‐up across the peer‐enhanced HCV services (*p* > 0.05).
[35]	Keats et al. (2015)	Australia	Community	Mixed methods	To evaluate assessment and treatment services for HCV infection among PWID attending a clinic integrating HCV and peer‐based support within an existing OST clinic.	**Peer workers:** not reported. **Intervention recipients:** *n* = 1447, gender not reported, mean age 40 years, age range not reported.	Peer workers as minor part of a larger HCV intervention	HCV education (patient), Recruitment/Outreach, health education, advocacy, treatment support.	The peer intervention facilitated HCV engagement and was financially sensible; peer workers freed up HCV nurse specialists' time. Peer workers were more likely to spend > 60 min with clients to engage, educated and supported clients (30% vs. 4% with an HCV nurse specialist), while HCV nurse specialists were more likely to spend 15‐30mins with clients on specific topics such as HCV treatment and pathology tests (42% vs. 22% with a peer worker) (*p* < 0.001).
[36]	Levander et al. (2021)	USA	Community	Qualitative	To explore patient perspectives of potential hospital‐based peer interventions for improving access to HCV treatment for PWUD. This is an extension of an existing peer intervention framework within the study setting.	**Peer workers:** not reported. **Intervention recipients:** *n* = 27, male *n* = 18 (67%), mean age 41 years, age range not reported.	Peer workers as minor part of larger HCV intervention	Treatment support, self‐disclosure, harm reduction, emotional support, hospital based.	Intervention recipients reported that peer worker support in hospital‐based HCV interventions could be both a barrier and a facilitator to engagement. Participants highlighted the importance of personal choice regarding peer worker support.
[37]	Maclellan (2017)	UK	Community	Qualitative	To explore how peers achieve their connections to facilitate the wellbeing and health service engagement of their clients.	**Peer workers:** *n* = 5, gender not reported, mean age 48 years, age range not reported.	Peer workers as main intervention Peer workers as research participants	Therapeutic alliance, emotional support.	Peer workers reported to use techniques to establish a positive therapeutic alliance: ‘rapport’, ‘self disclosure’ and ‘shared group membership’.
[38]	Midgard et al. (2021)	Norway	Community	Quantitative	To evaluate HCV treatment uptake and associated factors among HCV RNA positive PWID following POC testing and liver disease assessment in a peer‐driven decentralised mobile HCV clinic.	**Peer workers:** not reported. **Intervention recipients:** *n* = 305, male (77%), mean age 51 years, age range not reported.	Peer workers as main intervention	Assessment and data collection, outreach.	Peer workers facilitated patient engagement and HCV treatment uptake. HCV case finding was 34% (*n* = 102/296). Treatment uptake within 6 months was 88% (*n* = 90/102). [No comparison group]
[39]	Nagot et al. (2021)	France	Community	Quantitative	To assess whether an innovative community‐based, respondent‐driven sampling (RDS) survey, coupled with HCV screening and immediate treatment, is efficient to detect and cure current PWUD with chronic HCV.	**Peer workers:** *n* = 5, no demographics reported. **Intervention recipients:** *n* = 554, male 79%, median age 39 years, age range not reported.	Peer workers as minor part of larger HCV intervention	Treatment support, emotional support.	Peer workers contributed to HCV case finding, screening and support provision during HCV treatment. New HCV cases were identified at 32.6% (*n* = 181/554). Of these 8.8% (*n* = 49/181) were HCV RNA positive, and 75.5% initiated treatment (*n* = 37/49). The completion rate was 81.1% (*n* = 30/37) and 73% (*n* = 27/30) achieved SVR‐12. Costs per person screened was 161 EUR. There was no comparison group, but the authors suggested screening was cost‐effective when compared to the HepCare project (194–635 EUR).
[40]	Noller et al. (2020)	New Zealand	Community	Quantitative	To ascertain the viability of rapid POC testing for HCV antibodies by non‐clinical frontline needle and syringe staff (peer workers).	**Peer workers:** not reported. **Intervention recipients:** *n* = 204, male 66%, mean age 43 years, age range not reported.	Peer workers as main intervention	Testing, data collection, emotional support, provision of results, engagement.	Peer workers facilitated HCV testing uptake. Of *n* = 204 people tested, 64.2% (*n* = 131) were HCV antibody positive. Overall, 25.4% untreated HCV RNA were identified (*n* = 14/55). Of these, 50% commenced HCV treatment (*n* = 7/14). [No comparison group]
[41]	Pandey et al. (2021)	Canada	Community	Mixed methods	To develop a community‐led model to address HCV in community and report on the outcomes of screening events.	**Peer workers:** *n* = 5, male 60% (*n* = 3) in focus groups/interviews; total number of peers delivering intervention not reported. **Intervention recipients:** *n* = 540, no demographics reported.	Peer workers as minor part to larger HCV intervention Peer workers as research participants	HCV education (patients), recruitment and engagement, intervention design, indigenous, promotion, mobile.	Qualitatively, peer workers contributed to the development, design and implementation of the community‐led HCV care model. Quantitatively, 41.7% (*n* = 120/294) were tested HCV RNA positive. New undetected HCV diagnoses were 11.7% (*n* = 14/120), and linkage to care was 88% (*n* = 45/51) among those with chronic HCV (*n* = 51/120). [No comparison group]
[42]	Roose et al. (2014)	USA	Community	Mixed methods	To describe an innovative HCV Peer Educator programme that facilitates education, support and engagement in HCV treatment among patients in an opioid treatment programme.	**Peer workers:** *n* = 17, male *n* = 13 (76%), mean age not reported, age range: 34‐64 years.	Peer workers as main intervention	HCV education (patients), role modelling, treatment support, recruitment/outreach, group facilitation.	Of 76 intervention recipients who initiated HCV treatment through the peer intervention, *n* = 49 received HCV DAAs. [No comparison group] Qualitatively (authors' reflections), facilitators to the implementation of the peer intervention were: (1) Collaboration, (2) Co‐facilitation, (3) Peer selection. Barriers to the implementation of peer intervention were: (1) Ensuring confidentiality, (2) Establishing clear boundaries and roles, (3) Transition opportunities.
[43]	Stagg et al. (2019)	UK	Community	Quantitative (RCT)	To assess the efficacy of a community‐controlled, individual‐level, peer support intervention to promote engagement with healthcare services in individuals chronically infected with HCV.	**Peer workers:** *n* = 7, no demographics reported. **Intervention recipients:** *n* = 101, male 76%, mean age 43 years, age range not reported.	Peer workers as main intervention	Treatment support, engagement, acted as a permanent address for appointment letters, other health issue support, signposting (housing, benefits), self‐disclosure.	The peer intervention statistically increased the likelihood of a successful HCV treatment outcome in patients. (36.5% with peer workers vs. 18.4% in standard care) (18.1%, 95% CI 1.0%–35.2%, *p* < 0.05).
[44]	Surey et al. (2021)	UK	Urban	Qualitative	To chart the experience of peer workers within HepCare.	**Peer workers:** *n* = 5, male *n* = 5 (100%), mean age not reported, age range: 32–60 years.	Peer workers as main intervention Peer workers as research participants	HCV education (staff), BBV testing and pre‐treatment assessments, advocacy.	Peer workers reported their experience of transitioning to advanced peer support worker; themes were: (1) Transition to Integration (roles and responsibilities, inclusion in teams), (2) Retaining ‘Peerness’ (lived experience, empathy, need for support) and (3) Practising Critical Resilience (lived experience, emotional stability).
45	Thornton et al. (2018)	New Mexico	Prison	Mixed method	To describe the peer intervention (ECHO) impact on peer workers and intervention recipients.	**Peer workers:** *n* = 482, male *n* = 463 (96%), mean age not reported, age range: 26–45 years. **Intervention recipients:** *n* = 1113, male *n* = 564 (51%), mean age not reported. Age range: 25–45 years.	Peer workers as main intervention Peer workers as research participants	HCV education (patient), data collection, harm reduction, recruitment.	The peer intervention training statistically increased scores in HCV knowledge (*p* < 0.01), attitudes (*p* < 0.001), behavioural intentions (*p* < 0.001) and self‐efficacy (*p* < 0.001) in peers (pre/post training). The peer intervention statistically increased scores in HCV knowledge (*p* < 0.001) and behavioural intentions (*p* < 0.01) in intervention recipients pre/post training by peers. [attitudes and self‐efficacy not reported] Qualitatively, peer workers reported their experience as positive due to perceptions of [themes] (1) *(increased)* Respect from peers and prison staff, (2) *(increased)* self‐efficacy, confidence and self‐esteem, (3) *(commitment to)* Sharing information inside and outside of prison and (4) *(responsibility)* Passion to help.
46	Tookey et al. (2018)	Canada	Community	Qualitative	To gain an in‐depth understanding of the facilitators and challenges in the transition from client to support worker within a multi‐disciplinary, primary care based HCV treatment programme.	**Peer workers:** *n* = 2, male *n* = 1 (50%), mean age 49 years, age range: 48‐50 years.	Peer workers as main intervention Peer workers as research participants	HCV education (patient), self‐disclosure, emotional support, treatment support, administrative support, training peer workers.	Peer workers reported on the experience of transitioning from client to peer worker. [themes] (1) ‘the role of prior experience’, (2) ‘change in substance use practices’, (3) ‘shifts in relationships with community members and friends’, (4) ‘supportive organisational and structural factors’ and (5) ‘role transition as a journey’.
47	Treloar (2018)	Australia	Community	Mixed method	To examine the consistency and acceptability of a peer‐supported HCV and STI intervention.	**Peer workers:** not reported. **Intervention recipients:** *n* = 732, male *n* = 353 (53%), mean age 36 years, age range: 14‐91 years. **Qualitative** **Intervention recipients**: *n* = 19, no demographics reported **Health care staff:** *n* = 13, no demographics reported.	Peer workers as main intervention	HCV education (patients), health education (patients), Incentives, indigenous, recruitment.	The inclusion of Aboriginal peer workers at all intervention sites contributed to a significant increase in patient engagement (site 1: 11% to 52%, site 2: 5.9% to 8.4%, *p* < 0.001 for both). Qualitatively, intervention recipients reported facilitators to the peer‐led intervention's success. [themes] (1) ‘a stigma free health care and education program led by aboriginal workers’, (2) ‘incentives’ (staff perceived barrier), (3) ‘workforce capacity’ (staff perceived barrier), (4) ‘management strategy’. Healthcare staff also considered (1) ‘a stigma free healthcare and education program’ and (4) ‘management strategy’ as positive, however, they considered (2) Incentives and (3) Workforce (resources) as barriers.
48	Treloar et al. (2016)	Australia	Community	Qualitative	To examine Aboriginal Australians' experiences of, and decisions about, diagnosis, care and treatment for HCV.	**Peer workers:** not reported. **Intervention recipients:** *n* = 39, male *n* = 23 (59%), mean age 40 years, age range: 21–60 years.	Peer workers as minor part of larger HCV intervention	HCV education (patient), treatment support, self‐disclosure, support with peripheral issues, Indigenous peers.	Eleven participants had received peer worker support. Among these, the peer intervention was reported to be helpful in participants' decision making to initiate HCV testing and/or treatment via peers' sharing of their own experience of treatment.
49	Treloar et al. (2015)	Australia	Community	Qualitative	To examine the performance of two (newly established) community‐controlled peer support services from client, staff and peer worker perspectives.	**Peer workers:** *n* = 3, no demographics reported. **Intervention recipients**: *n* = 31, male *n* = 20 (66%), mean age not reported, age range: 25–58 years. **Health care staff:** *n* = 8, no demographics reported.	Peer workers as main intervention Peer workers as research participants	HCV education (patient), advocacy, Treatment support, emotional support.	All participants reported that peer workers had contributed to facilitating improved access to HCV testing and treatment, in particular via providing social and emotional support and additional information. In addition, positive changes were attributed to the peer intervention including enabling access to additional clinical and social support services (e.g., dental care), peer workers as mediators, clinical atmosphere more positive and client‐friendly.
50	Ward et al. (2019a)	USA	Community	Quantitative (RCT)	To test if novel interventions of cash incentives and peer mentors increase the rate of HCV treatment initiation and cure in a population of persons with HIV who use drugs (PWUD) compared to usual care.	**Peer workers:** not reported. **Intervention recipients:** *n* = 144, male 61%, mean age and age range not reported.	Peer workers as main intervention	Treatment support, incentives.	Regardless of intervention, there was no association with increased HCV treatment uptake (*p* > 0.05). Descriptively, the peer intervention resulted in a higher HCV treatment uptake when compared to usual care and cash incentives (83% vs. 76% and 67% respectively).
51	Ward et al. (2022)	USA	Community	Quantitative (RCT)	To examine ‘persistence’ with HCV treatment course and adherence to HCV medication, and their relationship with SVR among persons with HIV, HCV and SUDs. RCT groups: (1) usual care, (2) peer support, (3) cash incentive.	**Peer workers:** not reported. **Intervention recipients:** *n* = 110, male 59%, median age 55 years, age range not reported.	Peer workers as main intervention	Recruitment, engagement and treatment support, after care.	Regardless of intervention, there was no association between HCV treatment adherence and SVR (*p* > 0.05) meaning that nonadherence did not necessarily prevent achieving SVR.
52	Ward et al. (2019b)	UK	Community	Quantitative	To assess the cost‐effectiveness of the HepFriend initiative based on *n* = 89 HCV treatments over 3 years.	**Peer workers:** not reported. **Intervention recipients:** not reported.	Peer workers as main intervention	Treatment support, outreach, engagement and recruitment.	When compared to standard care, and using a 50 year time horizon, the added peer intervention HepFriend, i.e., a mobile HCV van with peer support for people who are homeless and inject drugs, was cost‐effective. The costs of the intervention were analysed using the number of HCV infections and deaths averted due to the intervention.
53	Bonnington et al. (2017)	UK	Community	Qualitative	To inform and assess the HCV peer support implementation in drug treatment settings, with a focus on the peer education and buddy support component.	**Peer workers**: not reported. **Intervention recipients:** *n* = 48, no demographics reported. **Drug treatment service providers:** *N* = 48, no demographics reported.	Peer workers as main intervention Peer workers as research participants	HCV education (patients and staff), self‐disclosure, harm reduction, emotional support, treatment support, family liaison.	Barriers to implementing peer workers into services were organisational, cultural (‘recovery’ focussed) and lack of support within services. Intervention recipients reported they felt ‘let down’ by the peer intervention as peer workers were governed by services/organisations, which did not align with the expectations of intervention recipients (who wanted peer workers outside organisational roles).
